# The Prophylactic Use of Bovine Colostrum in a Murine Model of TNBS-Induced Colitis

**DOI:** 10.3390/ani10030492

**Published:** 2020-03-15

**Authors:** Laura Menchetti, Giulio Curone, Iulia Elena Filipescu, Olimpia Barbato, Leonardo Leonardi, Gabriella Guelfi, Giovanna Traina, Patrizia Casagrande-Proietti, Federica Riva, Anna Beatrice Casano, Federica Piro, Daniele Vigo, Alda Quattrone, Gabriele Brecchia

**Affiliations:** 1 Department of Veterinary Medicine, University of Perugia, Via San Costanzo 4, 06126 Perugia, Italy; laura.menchetti7@gmail.com (L.M.); olimpia.barbato@unipg.it (O.B.); leonardo.leonardi@unipg.it (L.L.); gabriella.guelfi@unipg.it (G.G.); patrizia.casagrandeproietti@unipg.it (P.C.-P.); annabeatrice.casaro@libero.it (A.B.C.); alda.quattrone@hotmail.it (A.Q.); 2 Department of Veterinary Medicine, University of Milano, Via dell’Università 6, 26900 Lodi, Italy; giulio.curone@unimi.it (G.C.); federica.riva@unimi.it (F.R.); daniele.vigo@unimi.it (D.V.); 3 L’Arca Veterinary Clinic, Viale Antonio Gramsci, 141/E, Cortona, 52044 Arezzo, Italy; filipescu.iuliaelena@yahoo.com; 4 Department of Pharmaceutical Sciences, University of Perugia, Via A. Fabretti 48, 06123 Perugia, Italy; giovanna.traina@unipg.it; 5 Department of Chemistry, Biology and Biotechnology, University of Perugia, Via Elce di Sotto 8, 06123 Perugia, Italy; piro.chica@gmail.com

**Keywords:** bovine colostrum, TNBS-induced colitis, TLR4, cytokines, microbiota

## Abstract

**Simple Summary:**

Colostrum is the first milk secreted by the mammary glands, and it is very rich in bioactive components. Recently, the importance of bovine colostrum (BC) as a nutraceutical product has been emerging with regards to gastrointestinal diseases. One of the most widespread gastrointestinal disorders is the inflammatory bowel disease (IBD), a multifactorial chronic condition that has a powerful impact on the social life of millions of people. Because current therapy protocols neither ensure complete recovery from IBD nor are free of secondary side effects, the present study assessed the impact of a short-term prophylactic oral administration of BC in a murine model of TNBS-induced colitis. BC administration was both well tolerated and did not induce any pathological symptoms. It considerably modulated the response to inflammation through modifications of the TLR4 and cytokines gene expression profiles as well as that of the intestinal microbiota. Although further studies are needed to develop a precise therapeutic protocol of BC administration, it seems to have the potential to be used as a natural supplement in the treatment of IBD.

**Abstract:**

This study investigated the effects of a short-term administration of bovine colostrum (BC) in a TNBS model of induced colitis. Colitis was induced by TNBS treatment after seven days of BC (BC group, n = 12) or saline (control group, n = 12) administration in mice. Clinical signs, histopathological characteristics, expression levels of Toll-like receptor 4 (TLR4), pro- and anti-inflammatory cytokines, and microbial composition were assessed. BC was well tolerated and did not induce any histological damage or clinical symptoms. After TNBS treatment, the BC group showed a reduction in body weight (BW) loss compared to Control (*p* < 0.05). Moreover, expression levels of TLR4 (*p* < 0.01), Interleukin-1β (IL-1β; *p* < 0.001), Interleukin-8 (IL-8; *p* < 0.001), and Interleukin-10 (IL-10; *p* < 0.001) were lower in mice administered with BC. Finally, *Escherichia coli* were higher (*p* < 0.05), while Enterococci (*p* < 0.001), *Lactobacillus* spp. (*p* < 0.001), and *Bifidobacterium* spp. (*p* < 0.05) were lower in Control than BC group. This study confirms that pre-treatment with BC modulates the expression of genes and the count of microbes involved in the etiopathogenesis of colitis.

## 1. Introduction

Colostrum is the first milk secreted by the mammary glands of mammals in the first 3–4 days after giving birth, before it gradually transforms into mature milk. Colostrum of ungulates, especially bovine colostrum (BC), has high homology with humans and, compared to other animal species, it is richer in biologically active molecules which are essential for specific functions [1,2,3]. BC plays an essential role in maintaining a good health status and promoting the growth of the newborn given that it is rich in carbohydrates, proteins, fats, vitamins, and minerals. Moreover, it contains components that provide protection against pathogens (immunoglobulins, lactoferrin, lysozyme, lactoperoxidase), boost the maturation of the immune system (colostrinin, cytokines, lactoferrin, β-lactoglobuline, α-lactalbumin, and glycomacropeptide), and balance the intestinal microbiota (oligosaccharides, gangliosides and nucleosides). Finally, BC also contains growth factors that play important roles in the development, maturation, and repair of various tissues including the intestine [3,4,5,6]. Several studies and clinical trials, both in vitro and in vivo on humans and animals, suggest that these beneficial properties of colostrum can be exploited in the prevention and treatment of various gastrointestinal diseases [3,5,7,8].

One of the most widespread gastrointestinal disorder affecting millions of people worldwide is IBD [9]. IBD is a multifactorial, chronic, invalidating, and relapsing inflammation of the gastrointestinal tract. Although the etiology is still uncertain, the initiation, as well as the progression of IBD, is affected by the complex interaction of genetic, environmental, and immunological factors [9,10,11,12]. Therefore, in order to develop effective therapies for its treatment, it is essential to gain further insights into the etiology and pathogenetic mechanisms of IBD.

To date, an altered immune response is considered an important element related to the pathogenesis of IBD. Microbial recognition performed by immune and epithelial cells through the toll-like receptors is essential to begin the immune response and, at the same time, maintain a state of immune tolerance [13,14]. Specifically, toll-like receptors 4 (TLR4) are expressed on the surface of different intestinal cells and play a crucial role in several intestinal functions as well as in the host defense [14,15,16]. TLR4 recognizes lipopolysaccharides (LPS), which is the major component of the outer membrane of the cell wall of Gram-negative bacteria [15,16,17]. The interaction between TLR4 and LPS results in a cascade of intracellular signaling pathways leading to the activation of the nuclear factor kB (NF-kB) [13,14] and subsequently to the transcription of several genes involved in the host defense including those for the inflammatory cytokines, chemokines, and other inflammatory mediators [15,16,18].

The host tolerogenic response seems to be coordinated and mediated by several bacterial species of the intestinal microbiota, which could have a central role in the etiopathogenesis of IBD [9,19,20]. Indeed, a microbial imbalance causes a reduction of the intraluminal levels of butyrate, thus leading to an underexpression of certain tight junction proteins and increased epithelial permeability [21]. Epithelial barrier dysfunction increases bacterial translocation, a noted feature in IBD [9,19]. 

Current treatment for IBD is based on the administration of high-dose steroids, aminosalicylate, immunosuppressive agents, and antibiotics. However, these treatments are not medically effective and often surgery is required. Moreover, these drugs show strong side-effects that outweigh their benefits when used for long-term treatment [9,12]. 

In this context, BC has the potential to be considered a natural nutraceutical product that does not show adverse effects and that may be evaluated as an alternative or as a coadjuvant to the synthetic drugs in the prevention and treatment of different gastrointestinal diseases, including IBD [5,8,22]. Nevertheless, studies concerning the clinical use of BC in IBD are still limited, heterogeneous, and controversial; thus, further research is required before recommendations can be made for clinical application [7,23,24]. Moreover, because IBD is a multifactorial immune disorder, a multidisciplinary approach is required to understand the effects and mechanisms of BC supplementation. Filipescu et al. [25], obtained encouraging results by using a 21-day pre-treatment of BC in mice with TNBS-induced colitis. However, these results need to be confirmed, and the effects of a short-term preventive treatment were not investigated. 

Therefore, in this study, the effects of a short-term pre-treatment of seven days with BC were evaluated. We used a previously validated experimental model using a multidisciplinary approach and in vivo and in vitro measurements to evaluate both clinical and biomolecular changes in course of TNBS-induced colitis in mice.

## 2. Materials and Methods

### 2.1. Mice

Obtained from Harlan Laboratoires (Correzzana D’Adda, Milan, Italy), six-week old CD-1 mice (30.1 ± 1.41 g) were housed in controlled environmental conditions at a constant temperature of 21 ± 1 °C, 12/12 h light/dark cycle, and 55 ± 10% relative humidity, and were acclimatized for 10 days before any experimental procedure. Standard mice chow and water were administered ad libitum. All procedures relating to animal care and treatments were in accordance with the guidelines of the Italian regulations (Ministerial Declaration 116/92), as well as with the European Economic Community regulations (EU Directive 2010/63/EU for animal experiments). All efforts were made to minimize animal distress and to use only the number of animals necessary to produce reliable results. A loss of >20% of BW was considered as the criterion for humane endpoint; no mice reached this criterion. Experimental protocols were approved by the Ethical Committee for Animal Experimentation at the University of Perugia, Italy.

### 2.2. Bovine Colostrum

The milk formula per 100 g contained the following: protein 46 g, total fat 24 g, carbohydrates 20 g, sugars 20 g, sodium 400 mg, IgG 15–20 %, energy 500 kcal/2092 Kj. Bovine colostrum was obtained from the first 3–4 days of milking after parturition, kindly provided by Nutrasumma^®^ (Phoenix, Arizona, USA). The quality of the First Milking Colostrum Powder was ensured by low heat, low pressure, and dried freezing processes. 

### 2.3. Induction of Colitis 

The experimental protocol is schematized in Figure S1 (Supplementary Materials). Before the beginning of the experiment, 24 CD-1 mice were randomly divided into two major groups (n = 12/group): Control and Bovine Colostrum (BC) group. For seven consecutive days, in addition to the normal chow, animals of Control group received an oral administration of saline solution (0.6 mL per mouse), and BC group received the same volume of bovine colostrum (100 mg of colostrum powder dissolved in 0.6 mL of saline solution per mouse). Bovine colostrum solution was prepared just before the use, by vortexing the milk powder with saline solution until homogenization. Both solutions were administered directly into the stomach using a plastic feeding tube attached to a 1 mL syringe (2 biological instruments, Basozzo, Varese, Italy).

At the end of the treatment with saline solution or BC, six mice from each group were sacrificed to evaluate the effects of colostrum administration in healthy animals (Control pre-TNBS and BC pre-TNBS). In the remaining mice, colitis was induced by treatment with 150 µL of 1% TNBS solution, as previously reported [26]. Briefly, mice were fasted for 24 h, lightly anesthetized with isoflurane (Merial, Milan Italy) and then inoculated intrarectally via a catheter equipped with a 1-mL syringe. The catheter was lubricated and was advanced inside the anus for 3 cm before releasing the solution. Then, the mice were held in a vertical position with the head downwards for 1 min to ensure the distribution of the TNBS within the colon. Three days later, animals were sacrificed to evaluate the preventive effects of colostrum administration in induced colitis (Control post-TNBS and BC post-TNBS).

### 2.4. Clinical Evaluation of Colitis (Disease Activity Index, DAI) 

After TNBS treatment, mice were daily monitored for clinical symptoms (stool consistency, weight loss, and rectal bleeding) to attribute a 0–4 score (Table S1) as previously described [25,27]. 

### 2.5. Tissue Processing

Mice were suppressed by cervical dislocation. Each animal underwent a macroscopic evaluation of the gastrointestinal tract in a sterile environment, under a fume hood, in order to prevent the contamination of samples. The entire digestive tract from the esophagus to the anus was aseptically removed, afterwards the colon was individualized and opened longitudinally. The luminal content of the colon was preserved in sterile pre-reduced PBS and immediately frozen at −80 degrees for the microbiological and bio-molecular assays. Moreover, samples of colon tissue were washed with saline solution and then placed in 10% formalin for histological processing.

### 2.6. Histological Analyses and Scoring

Samples of colon fixed in neutral buffered 10% formalin were dehydrated in different concentrations of alcohol and cleared in xylene before being embedded in paraffin at 65° Celsius. Sections of 4–5 µm were cut, stained with Haematoxylin and eosin (H&E; Merck KGaA, Darmstadt, Germany), and examined under the light microscope, analyzing each sample on ten different fields in a blinded interpretation to avoid any bias.

The following parameters were taken into consideration: the extent of destruction of normal mucosal architecture, presence and grade of cellular infiltrate, and muscle thickening. A score ranging from 0 for normal to 3 for extended damage was given to each parameter. The final score could sum up to a total of 9 points [25].

### 2.7. Microbiota Analysis 

For the enumeration of *E. coli*, Enterococci, Anaerobes, *Lactobacillus* spp., and *Bifidobacterium* spp. colon samples were collected in sterile conditions, immediately placed into an anaerobic chamber, dissolved in sterile pre-reduced PBS, and a sterile stick was used to put 1 g of intestinal contents into a sterile test tube together with 2 mL 0.9% sterile saline solution. The stool was pressed and mixed in this solution and the tube was brought to volume (10 mL) with 0.9% sterile saline solution. Each sample (0.1 mL) was serially diluted via 10-fold dilutions. Starting from the lowest concentration, dilutions were plated and cultured on different media in triplicate using the spread plate method.

Chromocult agar and Bile Esculin Azide Agar were used for the enumeration of *E. coli* and Enterococci, respectively. All the plates were incubated at 37 °C, aerobically, for 24–48 h. Brain Heart Infusion agar, Mann Rogosa Sharpe agar (MRS), and modified MRS agar (0.3% (w/v) sodium propionate, 0.2% (w/v) lithium chloride, 0.05% (w/v) cysteine hydrochloride, and 5% (v/v) defibrinated sheep blood were used for the enumeration of total anaerobes, *Lactobacillus* spp. and *Bifidobacterium* spp., respectively. Anaerobic incubation was carried out in anaerobic jars (Oxoid) at 37 °C for 48–72 h. Anaerobic conditions were obtained using Anaerogen (Oxoid) and were checked using methyl blue strips as oxidation–reduction indicator. The number of colonies was counted, and all the data are expressed as CFUxlog/g.

### 2.8. Detection and Quantification of Gene Expression

For molecular analysis, the mRNA was extracted from 10 μm paraffin-embedded tissue sections adjacent to that observed to histological analyses using FFPE RNA Purification Kit (Norgen Biotek Corp., Ontario, Canada) according to the manufacturer’s instructions. In order to prevent genomic DNA contamination, the samples were treated with RNase-free DNase I Kit (Norgen Biotek Corp) following the manufacturers’ recommendations. RNA concentration was assessed using the Qubit RNA assay (ThermoFisher Scientific, Kandel, Germany) and it was stored at −80 °C until further investigation.

A quantity of 20 ng of total RNA was reverse transcribed with iSCRIPT cDNA (Bio-Rad, Hercules, CA, USA). To check for genomic DNA contamination, controls without reverse transcriptase were included, according to the producer’s guidelines.

QPCR analysis was carried out with 5 μL of a ten-fold diluted cDNA, 10 μL of ITAQ universal probes supermix (Bio-Rad, Hercules, CA), 1 μL of PrimeTime qPCR IDT (Integrated DNA Technologies, Coralville, Iowa, USA), and water to a final volume of 25 μL. The sequences of the probes used to detect gene expression levels are listed in Table S2. All PCR reactions had an initial incubation at 95 °C for 15 minutes, followed by 45 cycles at 95 °C for 15 seconds and 60 °C for 1 minute, during which fluorescence data were collected (Bio-Rad iCycler Real-Time PCR). Each sample was run in triplicate and the results were averaged. Sample amplification fidelity was verified by dissociation curves and agarose gel electrophoresis. PCR products were purified and sequenced using a QIAquick PCR Purification Kit (Qiagen Inc., Valencia, CA, USA). The relative expression genes were normalized to actin B reference gene levels.

### 2.9. Statistical Analysis

Changes in BW were analyzed separately before and after TNBS treatment using the Linear Mixed model. These models included animals and days as subject and repeated factors, respectively, and evaluated the effects of Time (six levels before and three levels after TNBS treatment), Group (two levels: Control and BC groups), and the interaction between Group and Time. BW on day 1 or day 7 was included as a covariate. Sidak adjustment was used for carrying out multiple comparisons. In order to analyze mRNA expressions and bacterial counts, the main effects of TNBS treatment (two levels: pre- and post- TNBS treatment) and Group (two levels: Control and BC groups) were evaluated. Diagnostic graphics were used for testing assumptions and outliers. Bacterial counts were expressed and analyzed as log10 CFU per gram of colon samples. Results were expressed as estimated marginal means ± standard error (SE), while raw data were presented in figures. Mann–Whitney test was used to assess DAI and histological scores. Values were expressed as median (Mdn) and interquartile range (IQR) or 95% confidence interval (95% CI). Statistical analyses were performed with SPSS Statistics version 23 (IBM, SPSS Inc., Chicago, IL, USA). Statistical significance occurred when *p* ≤ 0.05.

## 3. Results

### 3.1. Body Weight (BW) and Disease Activity Index (DAI) 

**Figure 1:**
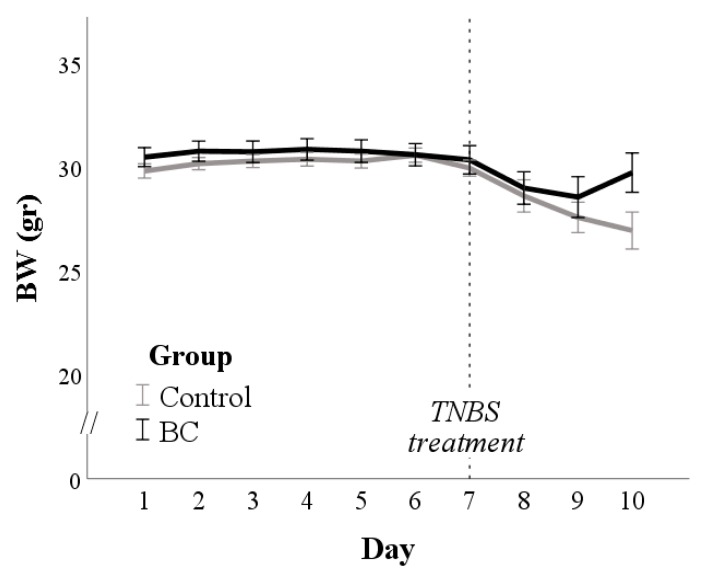
Bodyweight (BW) changes in control and bovine colostrum (BC) groups. The dotted line indicates the day of TNBS treatment.

The BW of mice from day 2 to day 7 was influenced only by their baseline BW (*p* < 0.001; Figure 1).

After TNBS treatment, 1 of 6 mice died within the control group, and mean BW was higher in BC than in the control group (*p* = 0.040; Figure 1). However, the DAI evaluated after TNBS treatment did not differ between the groups (Mdn = 2.3, IQR = 1.3–2.7 and Mdn = 1.2, IQR = 0.3–1.3 in Control and BC group, respectively; *p* > 0.1; Figure S2).

### 3.2. Macroscopic Evaluation

Gross lesions of the intestinal tracts show a different picture related to the examined groups. Animals treated with only tnbs didn’t show gross lesions to the colon tract. The mice group treated with tnbs without bc (control post-tnbs) revealed intense and diffuse hyperemia, sometimes congestion also associated with edema and thickening of long tracts of the intestinal wall. The group of mice treated with tnbs and bc (bc post-tnbs) showed grossly signs of mild inflammation essentially represented by edema although not always evident.

### 3.3. Histology

**Figure 2:**
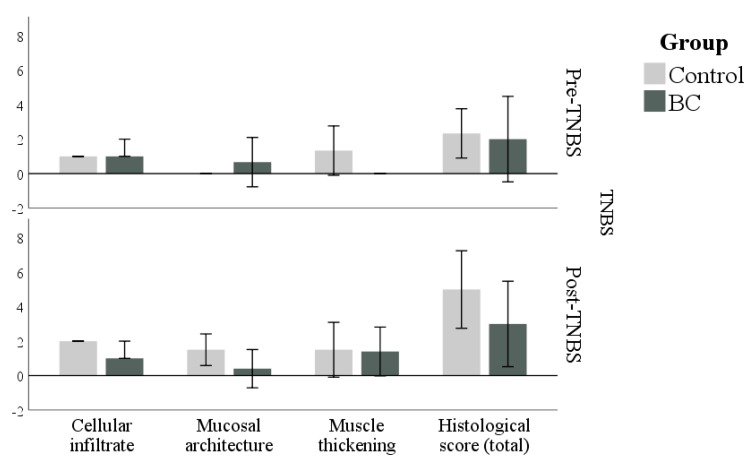
Scores for presence and grade of cellular infiltrate (Cellular infiltrate), the extent of destruction of normal mucosal architecture (Mucosal architecture), and Muscle thickening in addition to total score (histological score) in control and BC groups before (Pre-TNBS) and after (Post-TNBS) treatment with TNBS. No significant differences were found between the groups. Values are medians and 95% CI.

**Figure 3:**
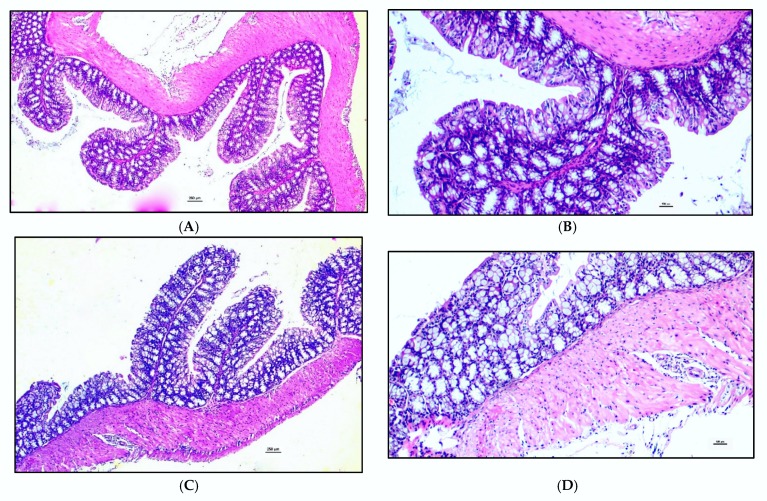
Histopathological findings regarding colon of mice treated for 7 days with Saline solution [(**A**) Control group pre-TNBS (4×), magnification 4×; (**B**) Control group pre-TNBS (10×), magnification 10×, histological score 3] or Bovine colostrum [(**C**) Bovine Colostrum group pre-TNBS (4×), magnification 4×; (**D**) Bovine Colostrum group pre-TNBS (10×), magnification 10×, histological score 1] before TNBS-treatment show that the signs of enteric inflammation are absent or very mild in both groups. Panels (**A**,**B**): mild to medium enteritis with evident inflammatory cell infiltrate of the superficial epithelium, mucosa and submucosa. Panels (**C**,**D**): very mild enteritis with light infiltration of lymphocytes in the focal distribution in the intestinal tracts.

The colon of BC and Control groups sacrificed before TNBS treatment had a low histological score (*p* = 0.700; Figure 2 upper panel). In particular, the signs of inflammation were absent or very mild in the colon of both groups (Figure 3).

After TNBS treatment, the histological score increased in the Control group (as a trend, *p* = 0.057), while it remained unchanged in the BC group (*p* = 0.571). However, the comparison between groups after TNBS treatment did not reveal any difference (*p* = 0.190; Figure 2, lower panel). As can be seen from the 95% CI, the histological scores had very variable values.

**Figure 4:**
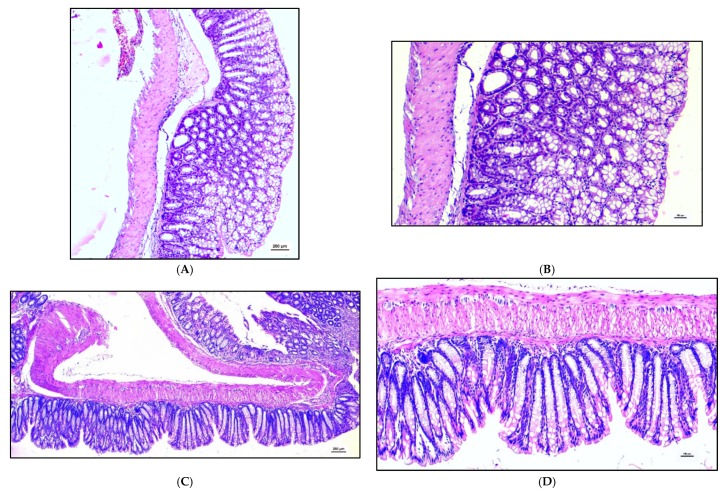
Histopathological findings regarding colon of mice treated for 7 days with Saline solution [(**A**) Control group post-TNBS (4×), magnification 4×; (**B**) Control group post-TNBS (10×), magnification 10×, histological score 6] or bovine colostrum [(**C**) Bovine Colostrum group post-TNBS (4×), magnification 4×; (**D**) Bovine Colostrum group post-TNBS (10×), magnification 10×, histological score 4] and then inoculated with TNBS show significant high but not always a constant amount of diffuse inflammation. The structure of the mucosa often appears modified by a massive diffuse inflammatory infiltrate of lymphocytes which involves also the submucosa and the intestinal wall. In the BC group, the diffuse inflammatory infiltrate of lymphocitary cells reaches the submucosa. Panels (**A**,**B**): enteritis with a high grade of diffuse inflammation and massive lymphocyte infiltration of all structures of the colon. The architectural structure of the mucosa is modified by the infiltration of the inflammatory cells. The submucosa, the intestinal wall and the serosal parts appear to be involved by the lymphocytary cells. Panels (**C**,**D**): enteritis with a medium grade of inflammatory infiltration involving the mucosa and the sub-mucosa of the colon.

After TNBS, severe lesions were observed in the colon of the control group with a significant high but not always a constant amount of diffuse inflammation. The structure of the mucosa appears modified by a massive diffuse inflammatory infiltrate of lymphocytes which also involves the submucosa and the intestinal wall. In the BC group, the diffuse inflammatory infiltrate of lymphocitary cells reaches to the submucosa (Figure 4). 

### 3.4. TLR4 and Cytokines Expression in Colon Evaluated by Real-Time PCR

Regardless of the group, treatment with TNBS increased the expression of TLR4 (*p* = 0.01), IL-1β (*p* < 0.001), IL-8 (*p* < 0.001), and IL-10 (*p* < 0.001). 

**Figure 5:**
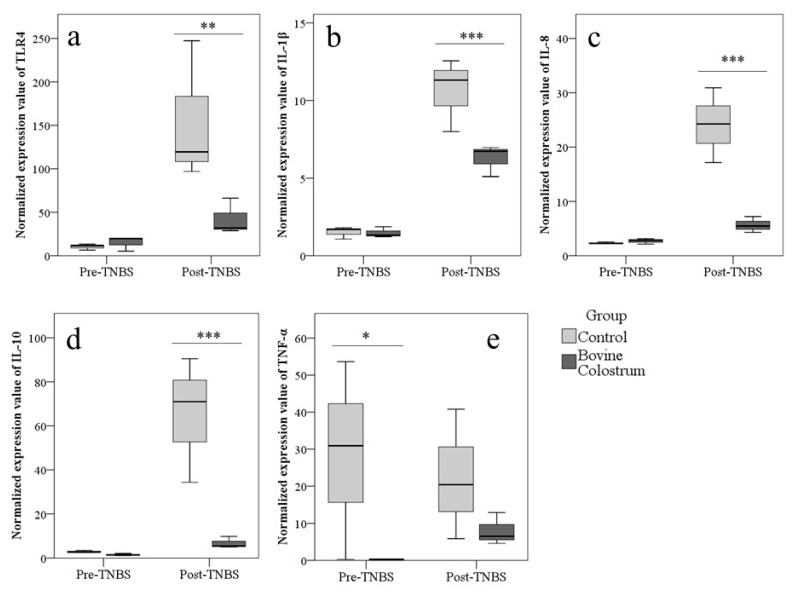
Preventive administration of bovine colostrum modulates the modifications of TLR4 and cytokines expression induced by colitis. Expression of TLR4 (**a**), IL-1β (**b**), IL-8 (**c**), IL-10 (**d**), and TNF-α (**e**) (2^−ΔCt^) in Control and Bovine Colostrum (BC) groups before and after treatment with TNBS in mice evaluated in colon by real-time PCR. * *p* < 0.05, ** *p* < 0.01, *** *p* < 0.001.

Before TNBS treatment, differences between groups were found only for TNF-α, with higher values in Control than BC group (*p* = 0.023; Figure 5). After TNBS, mice of BC group showed lower values of TLR4 (*p* = 0.002), IL-1β (*p* < 0.001), IL-8 (*p* < 0.001), and IL-10 (*p* < 0.001) expression (Figure 5).

### 3.5. Gut Microflora 

In the control group, TNBS treatment reduced Enterococci (−0.2 ± 0.1 log CFU/gr), *Lactobacillus* spp. (−0.6 ± 0.2 log CFU/gr), and *Bifidobacterium* spp. (−0.6 ± 0.1 log CFU/gr; *p* < 0.01), while *E. coli* and Anaerobes increased both in Control and BC group (+1.4 ± 0.5 log CFU/gr and +0.2 ± 0.1 log CFU/gr for *E. coli* and Anaerobes, respectively; *p* < 0.05; Table S3). 

**Figure 6:**
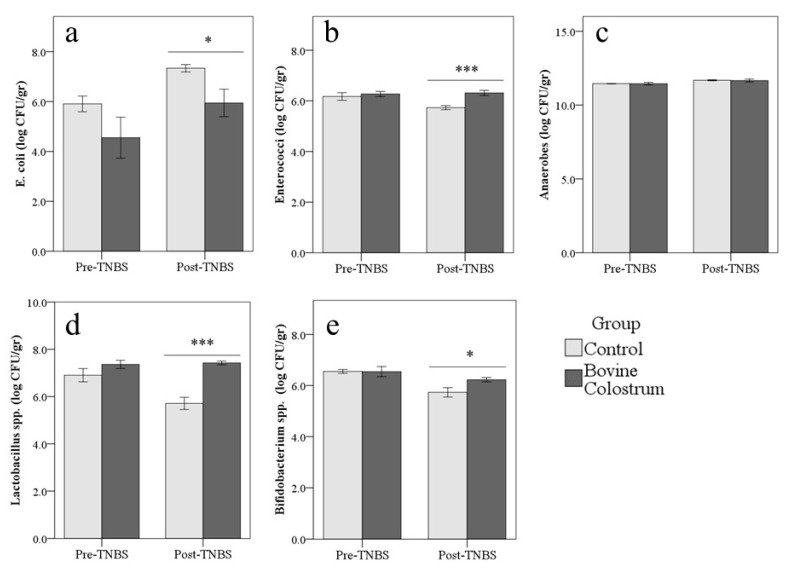
The preventive administration of bovine colostrum modulates the modifications of the microbial flora induced by colitis. Count of *E. coli* (**a**), Enterococci (**b**), Anaerobes (**c**), *Lactobacillus* spp. (**d**), and *Bifidobacterium* spp. (**e**) (log CFU/gr, Figure 3A–D and Figure 4A) in the control and BC groups before and after treatment with TNBS in mice. * *p* < 0.05, *** *p* < 0.001.

Pairwise comparisons showed that before treatment with TNBS, no significant difference was found between groups. Conversely after TNBS, count of *E. coli* (−1.4 ± 0.6 log CFU/gr; *p* = 0.023) was higher in Control than BC group, while Enterococci (+0.6 ± 0.1 log CFU/gr; *p* < 0.001), *Lactobacillus* spp. (+1.7 ± 0.2 log CFU/gr; *p* < 0.001), and *Bifidobacterium* spp. (+0.5 ± 0.1 log CFU/gr; *p* = 0.012) were higher in the BC group. Anaerobes were not influenced by bovine colostrum administration (*p* = 0.808; Figure 6).

## 4. Discussion

IBD are high-morbidity, multifactorial, chronic inflammatory diseases that affect millions of people worldwide and are associated with a decreased quality of life [10,12]. Crohn’s disease and ulcerative colitis are the major clinically defined forms of IBD [10]. Currently, the precise mechanisms involved in the pathogenesis of IBD are largely unknown, and a perfect model for their investigation does not exist. For these reasons, in recent years, many animal models and various chemical compounds have been employed to study its pathogenesis [8,26,28]. In our study, we designed the experimental procedures using the haptenic agent TNBS, which induces the disruption of the intestinal barrier and elicits a significant immune response that resembles IBD in regard to clinical symptoms and histopathology [25,26].

Moreover, the development of medically effective novel therapeutic approaches, based on the use of natural products with reduced adverse effects and low toxicity, for the long-term treatment of IBD is required [9,12]. A suitable candidate as adjuvant or alternative therapy is the colostrum [4,7,23,25]. Colostrum supplies a large number of biologically active compounds in a highly concentrated low-volume to ensure the growth and the protection of the neonate as well as favors the development and the maturation of several tissues and organs including the immune system and the gastrointestinal tract [1,3,5]. As previously reported, BC is from 100-fold to 1000-fold more potent than human colostrum given that it contains higher concentrations of some specific bioactive substances [1,2]. Furthermore, these compounds preserve their biological activity as they pass through the gastrointestinal tract leading to a beneficial effect on the intestinal functions [6,29]. This means that even humans and other animal species can count on BC to achieve health benefits [5,23]. There is emerging evidence that entire or fractioned BC and hyperimmune BC may become promising nutraceuticals that can prevent or alleviate various gastrointestinal disorders including IBD [4,7,8,23,24]. Thus, the present study was undertaken to assess the potential positive impact of a short-term prophylactic oral administration of BC in a murine model of TNBS-induced colitis. In agreement with the current literature [23,30], in the present study, we confirmed that BC is safe and well-tolerated by the mice given that the animals did not present any adverse effects, changes in body weight, damage or clinical signs during the experimental period before the treatment with TNBS. 

Administration with BC for seven days rather reduced BW losses after TNBS treatment, although it did not improve DAI and histological score. In the present study, the results of the clinical and histological scores were inconsistent, probably due to a high variability of the data. This result could also suggest that, unlike the long term, a short-term administration does not affect these aspects. Previous studies report conflicting results, depending on protocols and experimental models. Furthermore, it is difficult to find comparisons with our results because many of the other studies evaluated the therapeutic effect of BC and used the DSS to induce colitis which provides a different administration protocol compared to TNBS. Ya’acov et al. [30] administered colostrum or hyperimmune colostrum enriched with anti-*E. coli* LPS antibodies to mice for 4 days after treatment with TNBS and then sacrificed the animals. They obtained improvements of DAI and symptoms by using hyperimmune colostrum, on the other hand, the administration of raw BC did not show any significant amelioration on TNBS-induced colitis in mice. Conversely, other authors [8,24] have recently reported that also colostrum from nonimmunized cows can reduced BW losses, DAI and histological score in DSS-induced murine models of IBD. In humans, the administration of BC by enema for 4 weeks improved the clinical and histological score in patients affected by active colitis compared to control [7]. Finally, Filipescu et al. [25] evaluated the preventive effect of BC, however with a long-term treatment (21 days before the induction of the colitis) demonstrating that the administration of BC was able to reduce not only BW losses after TNBS treatment but also DAI and histological score in CD1 mice. Probably, it could be suggested that the effects of the BC supplementation are time-dependent, and at least 3 weeks of preventive treatment with BC are needed to achieve clinical improvements and counteract the inflammatory process induced by TNBS in the colon of mice.

However, other significant outcomes emerged from the data. First, the TNBS-induced colitis caused an alteration of the intestinal microbiota consisting in the increase of *E. coli* and in the reduction of both *Lactobacillus* spp. and *Bifidobacterium* spp. populations. Although the pathological mechanisms that induce the IBD remain essentially widely unknown and many factors seem to be involved [10], the alteration of gut microbiota seems to have an important role [9,20]. There is evidence that host intestinal immunological homeostasis is heavily influenced by the microbiota with the epithelium and underlying immune cells which normally perceive commensal microbes and mutually establish an immunologically tolerant mucosal barrier [9,19]. It is speculated that IBD results from an excessive and aberrant immune response to a normal commensal flora in genetically predisposed hosts, or a normal immune response to an abnormal intestinal microbiota [20,31]. Our findings seem to support the latter hypothesis. Importantly, this imbalance of microbiota was completely counteracted by the preventive administration of BC, which limits the reduction of the beneficial bacteria species and the increase of *E. coli* after TNBS treatment. Similar results were obtained after 3 weeks of BC administration [25]. In accordance with our results, Sugiharto et al. [32] found that piglets fed with BC for a week showed decreases in *E. coli* colonisation of the intestine. The intake of BC for 6 days improves microbial composition even in piglets submitted to chemotherapy [33]. On the other hand, Rasmussen et al. [34] reported no detectable difference in gut bacterial composition in preterm piglets treated with BC for 11 days. However, in light of our results, it seems that BC has a conservative effect on the intestinal microbiota, especially regarding the beneficial bacteria, even after short treatments. We can thus hypothesize that BC can stabilize intestinal microbiota favoring the elimination of dangerous bacteria such as *E. coli* and, at the same time, the growth of beneficial bacteria which in turn may contribute to reinforce the mucosal barrier function and to modulate the immune response reducing the severity of the inflammatory reaction.

TLR4 plays a crucial role in the maintenance of immune tolerance to commensal microflora [13,14]. They are expressed on the surface of the immune, epithelial, and stromal cells of the gastrointestinal tract [14,15]. TLR4 recognizes LPS and induce a series of events including the activation of NF-kB and the transcription of genes such as IL-1β, IL-6, IL-8, TNF-α, chemokines, nitric oxide, and cyclo-oxigenase-2 [18,28,35]. The present study confirms that the TLR4 and the cytokines secretion may have a pivotal role in the development of IBD. In fact, the TNBS induced colitis causes an increase in the expression of TLR4, IL-1β, and IL-8. Moreover, a remarkable finding of our study was that the preventive supplementation with BC specifically induced a down-regulation of the gene expression of TLR4 and proinflammatory cytokines after the treatment with TNBS compared to the control group in mice. In contrast, TNF-α showed a high variability in the expression. As TNBS-induced colitis model promotes a Th1 response, we expected a more marked increase in the TNF-α. Moreover, our study did not evaluate the expression of interferon-γ (INF- γ), another cytokine whose role in the TNBS model has been unexpectedly questioned [36]. Therefore, further studies are needed to clarify the responses of TNF-α and INF-γ. In light of this data, it seems that also a short-term BC pre-treatment remarkably attenuated the expression levels of TLR4, IL-1β, and IL-8. Thus it could be effective to prevent the activation of the innate immune system and of the resulting secretion of proinflammatory mediators in course of colitis in CD1 mice. These results are in accordance with Filipescu et al. [25] which, however, administered colostrum for a longer period before the induction of colitis. BC restores the intestinal function (increased villus height, galactose absorption, and brush-border enzyme activities) and reduces TLR4 and some inflammatory markers expression levels in the small intestine of a well established preterm pig model [37]. In agreement with our results, an inhibition of proinflammatory cytokines expression in a DSS model using Balb mice administered with colostrum whey and some components of milk after colitis induction was reported [24]. Conversely, in DSS-treated mice, Spalinger et al. [8] obtained the inhibition of the increase in *IL-6* and *IL-10* mRNA expression but not in INF- γ and TNF-α, with preventive administration of colostrum and hyperimmune colostrum. A previous study showed that a protein included in BC, the κ-casein-derived bovine glycomacropeptide is able to reduce the DAI, to lower the colonic damage score, and to switch off the inflammation inhibiting the NF-κB-mediated pro-inflammatory cytokine and other inflammatory markers expression [38]. An in vitro study in porcine intestinal epithelial cell exposed to pathogenic bacteria *E. coli* and *Salmonella enterica Typhimurium* indicated that BC whey protects the integrity of the intestinal mucosal barrier decreasing the expression levels of inflammatory genes and the activation of the NF-kB signaling pathway [39]. It was shown that BC has anti-inflammatory effects reducing the IL-1β and COX-2 expression through inhibition of NF-κB signaling pathway in human colon cancer cell line HT-29 [40]. In light of these data, it could be hypothesized that BC, rich in immuno-regulatory compounds may have modulatory effects in the intestinal immune system so that it may contribute to maintaining the immunological tolerance in the digestive tract. Moreover, the growth factors included in the BC may contribute to repair the mucosal lining of the gut favoring the remission of the clinical signs.

Treg and the products of their secretion, the anti-inflammatory cytokines IL-10 and Transforming Growth Factor-β (TGF-β) may play an essential role in the gastrointestinal homeostasis [10,41]. Indeed, there is evidence that IL-10 exhibits a suppressive and modulatory action by downregulating the activation of the immune cells and co-stimulatory molecules, thus protecting from immune-related mucosal injury during the chronic stage in IBD. Deficiency in IL-10 receptor [42], as well as changes in the subset and in the number of Treg in colonic mucosa and blood, induce the development of severe IBD in mice and humans [41,43]. As obtained using longer administration periods [25], in the current experiment, the treatment with BC for 7 days reduced the increase of IL-10 gene expression in TNBS-induced colitis in mice. Støy et al. [37] reported that IL-10 did not show any remarkable changes in preterm pig when administered with BC compared to the control group. Similar results were recently obtained by Spalinger et al. [8] after the administration of hyper-immune BC in DSS-treated mice. Conversely, Ya’acov et al. [30] reported that the oral administration of hyperimmune BC in mice, but not the raw colostrum, ameliorated the clinical signs of colitis, and this effect was associated with an increase in IL-10 blood concentrations as well as an increase in Treg. 

Both the expression of TLR4 and cytokines as well as the onset of the chronic inflammatory disease may be modulated by the complex microbiota harbored in the gastrointestinal tract [9,20]. Indeed, in the last decade, many researchers have confirmed that patients with gastrointestinal disease usually show a fecal and mucosa-associated imbalanced microbiota [9,44], and that in germ-free animals it is not possible to induce IBD [45]. The reduction of specific bacterial species could be responsible for the loss of immune tolerance given that it has been associated to a lower concentration of microbiome-derived LPS, in particular antagonistic forms of LPS, and to a decrease in butyric acid biosynthesis, which is able to show an immunoinhibitory activity, acting in the modulation of the TLR4 signaling [46]. Moreover, the changes in the microbiota composition may alter the expansion of Treg and then of anti-inflammatory cytokines production [46,47]. Finally, ineffective bacterial clearance due to the recognition of an impaired antigen in genetically predisposed individuals leads to a continuous TLR4 stimulation [14]. The disrupted mechanism of tolerance in epithelial and immune cells determines the secretion of proinflammatory cytokines and the activation of the adaptive immune system. This leads to a vicious cycle of aberrant immune response, mucosal inflammation, dysbiosis, and increased mucosal permeability, which would explain the persistent and recurrent nature of IBD [31,35]. 

For these reasons, the manipulation of the intestinal microflora may be a promising attractive therapeutic strategy to promote the growth of specific beneficial bacterial species able to counteract the dysbiosis, to modulate the immune system, and consequently to manage the IBD [9,12,29]. BC contains very high levels of oligosaccharides such as fructo-oligosaccharides and beta-galacto-oligosaccharides, gangliosides, and nucleosides which can act as prebiotics favoring the growth of a healthy colonic microbiota rich in *Bifidobacterium* spp. and *Lactobacillus* spp. that, in turn, modulates the immune system reducing the risk of the onset of an abnormal immune response [4,6,25,32].

## 5. Conclusions

In conclusion, seven days of administration of BC in TNBS-induced colitis provides time-manner promising results despite the short period of treatment without showing any adverse clinical signs or histological modifications. After TNBS treatment, clinical signs and histological scores are slightly influenced but BC seems to already work at the molecular level by modulating TLR4, pro-inflammatory cytokines, and microbiota. Data from the present study constitute an argument of using BC as complementary in the management of IBD. Moreover, they support the idea that dietary components, TLR4 activation, and intestinal microbiota may interact with playing a critical role in the IBD. The following studies will be needed in order to establish both the mechanisms that are behind IBD and the components contained in BC which may be responsible for the specific effects in the gut in order to develop a precise therapeutic or prophylactic protocol of BC administration.

## Supplementary Materials

The following are available online at https://www.mdpi.com/2076-2615/10/3/492/s1, Figure S1: Experimental Protocol, Figure S2: Percentage of weight loss, the score for stool consistency and rectal bleeding, and points of Disease Activity Index, Table S1: Disease activity index, Table S2: list of the PrimeTime qPCR, Table S3: Changes in bacterial counts (log10 CFU per gram of colon samples) after TNBS treatment for each group.
